# The Role of Exercise-Induced Arousal and Exposure to Blue-Enriched Lighting on Vigilance

**DOI:** 10.3389/fnhum.2018.00499

**Published:** 2018-12-10

**Authors:** Antonio Barba, Francisca Padilla, Antonio Luque-Casado, Daniel Sanabria, Ángel Correa

**Affiliations:** ^1^Centro de Investigación Mente, Cerebro y Comportamiento, Universidad de Granada, Granada, Spain; ^2^Centro de Estudios del Deporte, Universidad Rey Juan Carlos, Móstoles, Spain

**Keywords:** light, alertness, attention, circadian, temperature, physical activity, heart rate, ergonomics

## Abstract

It is currently assumed that exposure to an artificial blue-enriched light enhances human alertness and task performance, but recent research has suggested that behavioral effects are influenced by the basal state of arousal. Here, we tested whether the effect of blue-enriched lighting on vigilance performance depends on participants’ arousal level. Twenty-four participants completed four sessions (blue-enriched vs. dim light × low vs. high arousal) at 10 pm on four consecutive days, following a repeated-measures design. Participants’ arousal was manipulated parametrically through the execution of a cycling task at two intensities (low vs. moderate), and was checked by monitoring their heart rate. On each session, distal and proximal skin temperatures were recorded as a neuroergonomic index of vigilance, while participants performed a 20-min psychomotor vigilance task (PVT) under either blue-enriched light or dim light conditions. The Positive and Negative Affect Schedule (PANAS), Karolinska Sleepiness Scale (KSS), and Borg Rating of Perceived Exertion Scale (RPE) were used to measure subjective psychological state. The results showed that the exercise-induced manipulation of arousal produced robust alerting effects in most measures, while the lighting manipulation only attenuated subjective sleepiness and enhanced positive affect, but it did not influence behavior or physiology. Acute exposure to a blue-enriched light was practically ineffective when the arousal level was over baseline. The present research favored the use of acute physical exercise over acute exposure to blue-enriched lighting in order to boost humans’ alertness when necessary, as in work settings where maintaining optimal levels of attention is difficult (shift work, night-work, vigilance tasks) and necessary to prevent human error and accidents.

## Introduction

There consistent evidence supporting the alerting effects of light on subjective and physiological is measures (Cajochen et al., 2000, 2005; Lockley et al., 2006; Smolders and de Kort, 2014). However, this evidence becomes weaker at the behavioral level, as many studies have not confirmed that lighting effects improve task performance. A systematic review of the literature has revealed that from 17 studies testing the behavioral effects of bright white light as measured by a simple reaction time (RT) task, such as the psychomotor vigilance task (PVT; Dinges and Powell, 1985), only two of them reported significant effects on RT performance (Souman et al., 2017; see also Rodríguez-Morilla et al., 2018). Other studies using more complex tasks, like the n-back, have also failed to report behavioral effects of light (Vandewalle et al., 2007a,b).

While differences in task demands (i.e., type of cognitive processes involved, task difficulty) have been considered to explain the divergent results (Santhi et al., 2013; Gabel et al., 2015; Huiberts et al., 2015), other factors are likely mediating the finding of behavioral effects of light, for example, mental fatigue (Smolders and de Kort, 2014), defined as a biological drive for psychological restoration from prolonged or effortful cognitive activity (Williamson et al., 2011), and the level of basal vigilance just before light exposure (Correa et al., 2016). Vigilance is the cognitive ability to sustain attention over time to detect and respond to relevant stimuli during task performance (Warm et al., 2008). In Correa et al. (2016) study we measured basal vigilance through the PVT, and found that participants with higher vigilance (i.e., responded faster on the PVT) obtained a larger behavioral benefit from the blue-enriched light (vs. dim light) than participants with low vigilance in the Sustained Attention to Response Task (SART). However, this relationship between basal vigilance and light effects was found a posteriori and was based on correlational evidence (i.e., the effect of light on SART RTs was analyzed by controlling for individual differences in basal difference, including PVT performance as a covariate).

The aim of the current study was therefore to test experimentally the potential relationship between basal vigilance and behavioral effects of light by manipulating the arousal level parametrically. Arousal can be defined as the state of physiological activation regulated by the ascending reticular activating system, which mediates wakefulness and allows organisms a disposition to react to environmental demands; it can be measured through the heart rate (HR). Since higher arousal implies higher performance in easy cognitive tasks (Yerkes and Dodson, 1908) like the PVT, arousal manipulations would alter the vigilance level, eventually modulating the effects of light.

In fact, just a few studies had already manipulated the level of arousal by administering caffeine. Wright et al. (1997) examined whether the combination of bright light and caffeine mitigated the performance decrement caused by two nights of sleep deprivation. Lighting was applied throughout the night, whereas caffeine was administered at the beginning of the exposure to light. The results showed that both lighting and caffeine improved task performance, leading to the largest behavioral benefit when administered together. Beaven and Ekström (2013) further confirmed the interactive effects between blue light and caffeine on behavioral performance by exposing non sleep-deprived participants to 1 h of blue light. They found that the combination of light and caffeine increased response speed at the cost of decreased inhibitory control in a go-nogo task.

These studies altogether suggest that arousal increments produced by caffeine can potentiate the effects of light on behavioral performance. However, due to the pharmacokinetics of caffeine, the effects induced by its exogenous administration are not constant over time, which can make the control and continuous monitoring of arousal levels difficult. The present study aimed to address this issue by manipulating, for the first time, the physiological activation of individuals through acute physical exercise.

It is widely known that physical exercise elicits a variety of physiological changes at central and peripheral levels (e.g., HR, increases in core temperature, cortical blood flow, or plasma catecholamine concentration). Many of these responses have been linked to transient changes in the arousal state (Fisher, 2014; Nobrega et al., 2014), thus enabling the control and continuous monitoring of the arousal level throughout the duration of the treatment of interest.

The alerting effect of physical exercise can be further observed at the behavioral level, as a recent study showed that exercise of acute moderate-to-high intensity speeded responses in the PVT (González-Fernández et al., 2017). This behavioral effect is likely mediated by physiological arousal processes like increments in catecholamines, HR, lactate, and hormones of the hypothalamo-pituitary-adrenergic axis (Borer, 2003). Here we also used the PVT as the main behavioral task, thus facilitating comparisons between the effects of light and the effects of exercise. Another reason to use the PVT was based on our previous finding that blue-enriched light only modulated response speed but not response inhibition in the SART (Correa et al., 2016; see also Beaven and Ekström, 2013), suggesting that lighting effects would be most evident in simple RT rather than more complex tasks like the SART, which demands inhibitory control.

Therefore, in the present study we used an auditory version of the PVT to compare the acute effects of a blue-enriched light (vs. dim light) on behavior under high vs. low arousal conditions. The auditory PVT is commonly used in lighting research to measure the effects on the non-image forming system rather than on visual perception (Lockley et al., 2006). In addition to the behavioral effects of lighting, its impact was further measured at physiological and subjective levels.

To measure physiological effects of light we recorded skin temperature by attaching a small sensor to the skin at distal (wrist) and proximal (infraclavicular) sites, and computed the distal to proximal temperature gradient (DPG). This method is non-invasive, portable, wearable, and reliable (van Marken Lichtenbelt et al., 2006), then facilitating the collection of physiological measurements in natural environments and everyday settings. These features therefore make the recording of skin temperature a valid method in Neuroergonomics research. Research has revealed that increments in the DPG were associated with earlier sleep latency and higher somnolence at the beginning of the night (Kräuchi et al., 1999), and with lower task performance in vigilance tasks like the SART (Lara et al., 2018) and the PVT (Romeijn and Van Someren, 2011; Molina et al., 2017). Altogether, these findings suggest that skin temperature (mainly the DPG) could be used as a neuroergonomic marker of vigilance. This index is also sensitive to the alerting effects of light, as blue light exposure during the night decreases the DPG by activating sympathetic tone (Cajochen et al., 2005; Rodríguez-Morilla et al., 2017).

Finally, we used ambulatory circadian monitoring (ACM) to measure several markers of circadian rhythms, such as wrist temperature, motor activity, body position, and exposure to ambient light (Ortiz-Tudela et al., 2010), in order to obtain objective information on the sleep patterns of our sample throughout the 4 days of experiment.

On the basis of the research reviewed above, we expected main effects of both acute physical exercise and acute exposure to blue-enriched lighting on subjective and physiological markers of arousal (i.e., decrements in sleepiness and DPG temperature). Most relevant, the behavioral effects of lighting would depend on the arousal level induced by exercise, that is, we expected the largest increment in response speed on the PVT under the condition of blue-enriched light plus high exercise-induced arousal.

## Materials and Methods

### Participants

Twenty four subjects (19 females; *M* = 22.88; *SD* = 3.94) from the University of Granada participated in the study. Each participant completed a questionnaire about sleep, health habits and physical activities and the reduced Morning-Evening Questionnaire adapted to the Spanish population (rMEQ; Adan and Almirall, 1991) before the experimental sessions. The inclusion criteria were: having slept a minimum of 6 h prior to each of the four the experimental sessions, no intake of psychoactive substances, having intermediate chronotype, not practicing physical exercise for more than 2 h per week, no diagnosis of visual and other medical, psychiatric and sleep disorders, no current shift-work, and not having traveled to other time zones in the last 3 months. Due to procedural problems when recording circadian rhythms measures during the week (i.e., removal of the monitoring device at the time of sleep) one participant could not be included in analyses of sleep duration and time spent awake. The statistical analysis was performed with the collected data of 24 participants, except for circadian rhythms measures where we used the data of 23 participants. All participants gave prior written informed consent approved by the Ethics Committee of the University of Granada (REF: n.34/CEIH/2015) and they received 30 euro after the completion of the experiment for their participation. The anthropometric and physiological characteristics of the participants are presented in Table 1.

**Table 1:** Sleep variables, anthropometrical, and physiological scores (mean and standard deviation).

	*Mean*	*SD*
Age	22.88	3.94
Weight (kg)	67.17	13.90
Height (cm)	168.71	9.32
BMI (kg/m^2^)	23.61	4.68
HR Max (bpm)	191.99	2.76
rMEQ	14.83	1.66
MEQ	48.89	4.28
PSQI	7.46	3.09
Temperature (°C)	32.91	0.86
M5 Temperature (°C)	34.38	0.57
M5 (Hour)	4:54 am	1.58
L10 Temperature (°C)	31.76	1.33
L10 (Hour)	4:53 pm	2.39

*BMI, body mass index; HR, heart rate; bpm, beats per minute; MEQ, morningness-eveningness questionnaire; PSQI, Pittsburgh Sleep Quality Index; M5, midpoint of the five consecutive hours of maximum distal skin temperature; L10, midpoint of the 10 consecutive hours of minimum distal skin temperature. Maximum HR (HR Max) was estimated through a toolbox based on Tanaka et al. (2001) considering the following formula: HR Max = 208 – (0.7 x age).*

### Apparatus and Stimulus

#### Questionnaires and Scales

In the Positive and Negative Affect Schedule (PANAS; Watson et al., 1988), participants responded to two 10-item scales that measure both positive (e.g., excited, inspired) and negative (e.g., upset, afraid) affect. Each item is rated on a five-point scale ranging from *very slightly or not at all* (1) to *extremely* (5). The affective balance index is computed by subtracting positive minus negative affect scores, with higher scores representing a positive affective balance.

In addition to the reduced version (rMEQ), the Morningness–Eveningness Questionnaire (MEQ; Horne and Ostberg, 1976) was also administered to measure the participant’s chronotype. Scores can range from 16 to 86, including evening (16–41), intermediate (42–58), and morning chronotypes (59–86).

We used a general interview about health and sleep habits including questions about the day and night prior to the experiment (see Correa et al., 2016).

The Karolinska Sleepiness Scale (KSS; Akerstedt and Gillberg, 1990) measured the subjective level of sleepiness after exposure to each treatment condition, ranging from 1 (“totally alert”) to 9 (“maximum sleepiness”).

Subjective effects of physical exercise was measured by the Borg Rating of Perceived Exertion scale (RPE; Borg, 1970), ranging from *no exhaustion at all* (6) to *complete exhaustion* (20).

Sleep quality during the last month was measured through the Pittsburgh Sleep Quality Index (PSQI; Buysse et al., 1989).

Mental Effort related to the completion of the PVT was measured by a 10-point Likert scale.

Positive and Negative Affect Schedule and Borg RPE scales were administered in paper, while the remaining questionnaires were computerized and presented visually over a black background.

#### Lighting

**FIGURE 1:**
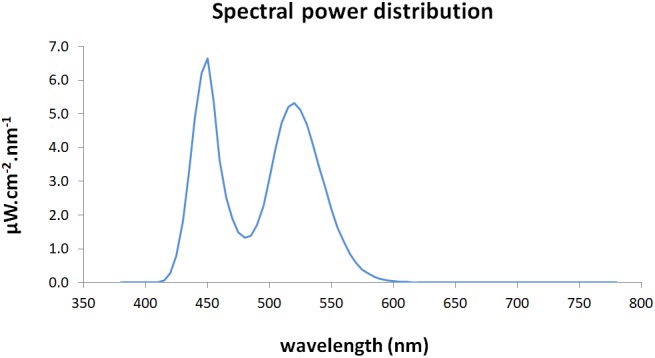
Spectral power distribution of the blue-enriched white light.

A matrix of 16 LEDs (*IgniaLight, SACOPA, S.A.U.*) emitted blue-enriched white light with a peak of 440 nm. Spectral power distribution was measured with an illuminance spectrophotometer (*KONICA MINOLTA; CL-500A*) at the eye level (Figure 1).

Illuminance measures for each of the retinal photoreceptors exposed to the blue-enriched light condition were estimated through the toolbox developed by Lucas et al. (2014; Table 2).

**Table 2:** Equivalent α-opic illuminance values for the blue-enriched light condition.

	λ_max_	α-Opic lux
Melanopsin	480.0	795.30
S-cone	419.0	980.87
M-cone	530.8	1088.84
L-cone	558.4	1118.04
Rods	496.3	924.58

In both lighting conditions the same lamp was turned on, but it was fully covered by an opaque box during the dim light condition. Their intensities were measured on each session, leading to averages of 1176.54 lx (*SD* = 60.55) and 0.44 lx (*SD* = 0.11) for blue-enriched and dim light, respectively.

#### Physical Exercise

A *VIAsprint 150 P* Cycle-ergometer (*Ergoline GmbH, Germany*) was used to manipulate exercise intensity individually, that is, monitoring of the HR on each participant served to adjust the scheduled level of physiological arousal (low vs. high). This adjustment was controlled by the experimenter, who manually adjusted the resistance of the cycle ergometer (Watts) in order to achieve and maintain the target HR estimated range for high (or low) arousal conditions (see section “Procedure” below for further details).

#### Physiological Recording

All participants wore a Polar V-800 monitor (Polar Electro Öy, Kempele, Finland) to record their HR [beats per minute (bpm)] during the experimental sessions. The elastic electrode transmitter belt (*POLAR H7* Bluetooth Smart sensor) was placed on the participants’ chest in the lower part of the sternum. The transmitter belt contains two electrodes to detect the voltage differential on the skin during every heartbeat and sends the signal continuously and wirelessly using an electromagnetic field to the *Polar V-800* receiver unit. The data were transferred to the Polar Flow web service (Polar Electro 2017) for further analysis.

Samples of body temperature were recorded every minute using a sensor (*iButton – DS1921H; Maxim, Dallas*), with a temperature range between +15°C and +46°C and 1°C of accuracy with a precision of 0.125°C. Sensors were placed on every participant with adhesive tape on the non-dominant wrist to record distal skin temperature, and on the left infraclavicular area to record proximal skin temperature. Room temperature variation was also recorded in every experimental session (ending minus initial temperature values) using a clock-thermometer (*Inovalley-F95310*).

During the days of the experiment, participants were continuously monitored using the ACM device KRONOWISE^®^ (*KW3*, *CRONOLAB, University of Murcia*). This wristwatch-like device measures body position, motor activity, distal temperature and ambient light (Ortiz-Tudela et al., 2010), providing an objective measure of circadian rhythms and sleep parameters of each participant during the week of the experiment.

#### Behavioral Task

The Auditory PVT was programmed through E-Prime 2.0 (Schneider et al., 2002) and run on a PC Intel Core 2, 17-inch computer screen. The target stimulus was a 700-Hz tone presented for 250 ms via Headphones (*Seinheiser HD 201*). Participants were instructed to respond as quickly as possible to the target onset, which appeared after an interval with a random duration between 2000 and 10000 ms on each trial. Task duration was 20 min.

### Design and Procedure

Participants first completed a brief interview (sleep habits, physical activity, and pharmacological treatment) and the reduced MEQ to check for inclusion criteria. Participants meeting the inclusion criteria completed the study over four consecutive nights at the same time of the day (starting either at 9 or 10 pm), differing in light condition (blue-enriched light and dim light) and exercise-induced activation (high arousal and low arousal). Participants were tested in all conditions (blue-enriched – high arousal, blue-enriched – low arousal, dim light – low arousal and dim light – high arousal), which order of presentation was partially counterbalanced across participants (ABCD, BCDA, CDAB, and DABC).

In the first session, the ACM device was placed on the participants’ non-dominant wrist and was kept throughout the 4 days of experiment. They were instructed to maintain a regular sleep-wake cycle and advised to avoid beverages containing caffeine, along with vigorous physical activity during the whole experiment. Anthropometric data [age, height, weight, and the body mass index (BMI)] were also collected during the first session.

At the beginning of each session, sensors were placed on distal (non-dominant wrist) and proximal (left infraclavicular) areas. Then, the participants completed the MEQ through the computer for the first 3 days and the PSQI in the fourth night. In a room adjacent to the experimental booth, they laid down on a mat for 10 min in total darkness to register their basal HR. Subsequently, we used the heart rate reserve index (HRR) for prescribing relative exercise intensity in the high arousal conditions (Mann et al., 2013). This method establishes the target HR that each participant must maintain during the experimental session to ensure the desired level of activation by taking into account both the maximal predicted HR (Tanaka et al., 2001) and the basal HR. A relative intensity of 60% of the HRR was set to induce the desired activation level in high arousal conditions. In the low arousal conditions, participants remained on the cycle-ergometer pedaling with no load and target HR below 25% of their individual HRR (see Table 3).

**Table 3:** Mean scores (and standard deviation) of controlled variables over the four experimental sessions.

Lighting	Blue-enriched		Dim	
Exercise-induced arousal	High	Low	High	Low
Room temperature variation	0.49 (0.04)	0.46 (0.04)	0.44 (0.04)	0.48 (0.04)
Basal temperature (°C)	32.89 (0.24)	32.77 (0.26)	33.06 (0.25)	32.48 (0.30)
Basal HR (bpm)	69.75 (2.00)	71.89 (1.89)	70.73 (2.15)	72.77 (2.42)
HR during PVT (bpm)	146.22 (0.91)	86.91 (2.16)	144.94 (2.12)	87 (2.40)
Target HR (bpm)	146.15 (0.98)	84.46 (1.73)	146.75 (1.07)	85.21 (2.26)
Reported sleep duration (hours)	7.66 (0.22)	8.17 (0.31)	7.12 (0.24)	7.43 (0.27)
Reported time awake (hours)	11.51 (0.30)	11.93 (0.33)	11.15 (0.27)	11.47 (0.37)

*HR, heart rate; bpm, beats per minute.*

**FIGURE 2:**
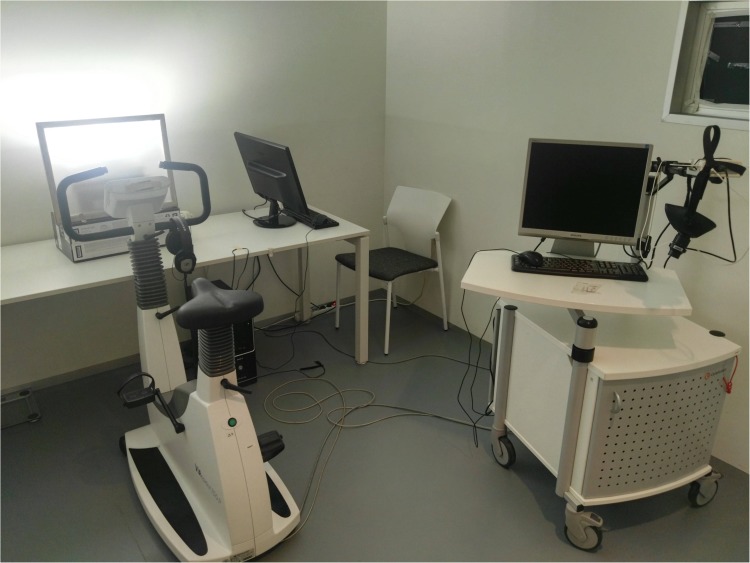
Experimental setup in the blue-enriched white light condition.

Once in the experimental room, they started a 20-min period of adaptation to the lighting condition, with the last 5 min pedaling as a way to adapt to the exercise-induced activation condition. Participants were seated in the cycle-ergometer in front of the LED lamp to adjust the height of the saddle and check for comfort (Figure 2). Distance to the lamp (about 70 cm) and intensity were measured for each participant.

**FIGURE 3:**
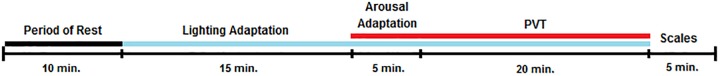
Overview of the experimental sessions. Black line: darkness; Blue line: lighting exposure; Red line: exercise-induced activation by pedaling; Scales: KSS (Karolinska Sleepiness Scale), PANAS (Positive and Negative Affect Schedule), RPE (Borg Rating of Perceived Exertion), and Mental Effort.

Then, the participants responded to the general interview whilst seated on the cycle-ergometer. Following the 20-min light adaptation period, they performed the auditory PVT for 20 min, wearing the headphones and responding with a push-button while they pedaled (Figure 3).

Participants were encouraged to maintain their eye gaze on a fixation point located on the wall throughout the adaptation period and task. During the arousal adaptation period and the PVT the experimenter adjusted the cycle-ergometer load to maintain the individual target HR previously estimated. Once the PVT was finished, the LED lamp was turned off and each participant completed the KSS, PANAS, Mental Effort, and Borg RPE scales in front of the computer. The participants were permitted to drink water before and after the cycle-ergometer test.

### Data Analysis

Repeated-measures analyses of variance (ANOVA) with Lighting (blue-enriched light and dim light) and Exercise-induced activation (high arousal and low arousal) as within-subject factors were performed on the following variables. Controlled variables were room temperature variation (final minus initial temperature), sleep duration and time awake (in hours, reported by the participants), HR and illuminance. Sleep duration was further estimated from the ACM data, by computing the duration where body position, ambient light, and physical activity were at their lowest and distal temperature was highest in the daily recordings, according to previous research using this system (Ortiz-Tudela et al., 2010).

Lighting × Activation ANOVAs were also performed on the following dependent variables: subjective somnolence from the KSS scores, both positive and negative affect and affective balance (positive minus negative affect) scores on the PANAS, subjective mental effort, perceived physical exertion through the Borg RPE scores, behavioral data from the PVT (mean RT, mean inverse RTs: 1/RT, and number of lapses: RTs > 500 ms; RTs below 100 ms were considered anticipations and excluded from this analysis) and physiological data from skin temperatures. Skin temperatures were computed using wrist and infraclavicular data during the PVT, corrected by a baseline during the 10-min rest period. The DPG was computed by subtracting distal minus proximal temperatures.

Given the elusive nature of lighting effects on behavior, a Bayesian ANOVA on PVT data was further performed to assess the probability of the results to fit the null hypothesis (i.e., to test further whether lighting really affects behavioral performance or not). We reported the Bayes factor (*B*_01_), the ratio of the probability of the data fitting under the null hypothesis vs. the alternative hypothesis. Bayes factors higher than 0.33 were interpreted as supporting the null hypothesis, being stronger with higher values. By contrast, values below 0.33 were considered as support to the alternative hypothesis, with lower values indicating stronger support (Jarosz and Wiley, 2014).

## Results

### Controlled Variables

No differences were observed between the sessions for room temperature variation and basal HR (all *ps* > 0.10; Table 3).

**FIGURE 4:**
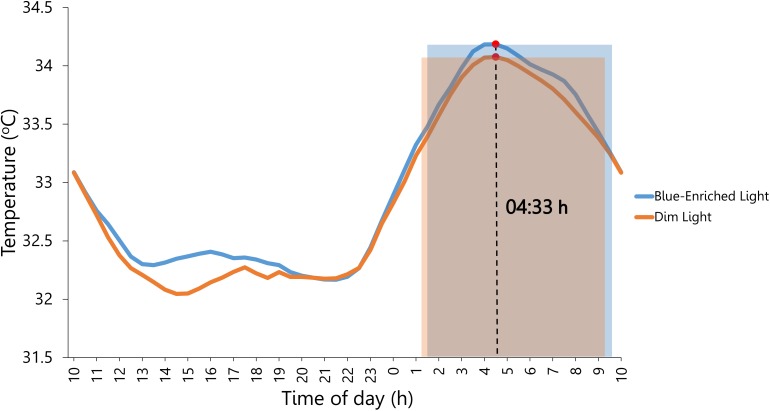
Circadian rhythm of wrist temperature averaged across participants prior to lighting exposure during the 4 days of the experiment. Blue and orange shaded rectangles: sleep period in the night before the exposure to blue-enriched light and dim light, respectively. Marked by a red circle (∙) is the distal temperature maximum value (M5), which estimated the midsleep time at 4:33 am.

Furthermore, we verified that both cycling conditions clearly produced differential exercise-induced activation during the PVT [high arousal: *M* = 145.58 bpm; *SD* = 1.29; and low arousal: *M* = 86.96 bpm; *SD* = 2.19; *F*(1,23) = 1098.88, *p* < 0.001, $\eta_{p}^{2}$ = 0.98]. Even though participants reported sleeping more before the blue-enriched light compared with the dim light condition, *F*(1,23) = 6.73, *p* = 0.02, $\eta_{p}^{2}$ = 0.23 (Table 3), the analyses on objective sleep duration using the data from Ambulatory Circadian Monitoring did not confirm an effect of Lighting (blue-enriched light: *M* = 8.11 h; *SD* = 0.24; and dim light: *M* = 7.99 h; *SD* = 0.24; *F* < 1; Figure 4). Therefore, although participants subjectively reported sleeping more in the blue-enriched condition (vs. dim light) they showed no objective differences in sleep duration.

### Subjective Measures

The Lighting (blue-enriched light and dim light) × Exercise-Induced Activation (high arousal and low arousal) ANOVA on Positive Affect (measured by PANAS) revealed higher scores after high arousal than after low arousal conditions [*M* = 35.83; *SD* = 1.10 and *M* = 32.63; *SD* = 0.97; *F*(1,23) = 5.64, *p* = 0.03, $\eta_{p}^{2}$ = 0.20]. The effect of *lighting* failed to reach statistical significance [blue-enriched: *M* = 34.98; *SD* = 0.88; dim light: *M* = 33.48; *SD* = 0.88; *F*(1,23) = 3.46, *p* = 0.08, $\eta_{p}^{2}$ = 0.13]. The interaction between *lighting and activation* was not significant either, *F* < 1. Negative affect was also higher after high arousal vs. low arousal conditions [*M* = 13.13; *SD* = 0.79 and *M* = 11.71; *SD* = 0.46; *F*(1,23) = 6.66, *p* = 0.02, $\eta_{p}^{2}$ = 0.22]. No other effects were significant (all *ps* > 0.10). Affective balance was higher in the blue-enriched (*M* = 22.98; *SD* = 1.07) than the dim light condition [*M* = 20.65; *SD* = 1.31; *F*(1,23) = 5.35, *p* = 0.03, $\eta_{p}^{2}$ = 0.19], indicating a prevalence of positive affect due to light. No other significant main effects or interaction were observed (all *ps* > 0.11).

Subjective Sleepiness (KSS) was lower after blue-enriched light vs. dim light conditions [*M* = 3.44; *SD* = 0.27 and *M* = 4.02; *SD* = 0.31; *F*(1,23) = 7.72, *p* = 0.01, $\eta_{p}^{2}$ = 0.25]. Additionally, subjective sleepiness was lower after high arousal than in low arousal conditions [*M* = 3.33; *SD* = 0.32 and *M* = 4.13; *SD* = 0.35; *F*(1,23) = 4.15, *p* = 0.05, $\eta_{p}^{2}$ = 0.15]. However, the interaction between lighting and activation was far from significant, *F* < 1.

The Lighting × Activation ANOVA on Mental Effort showed no effects involving the lighting factor (*Fs <* 1); only the main effect of activation was marginally significant, *F*(1,23) = 3.59, *p* = 0.07 (mental effort tended to be perceived as higher in high vs. low arousal conditions).

With regard to the Borg RPE scale, a main effect of *activation* revealed higher perceived physical exertion after high arousal vs. low arousal conditions [*M* = 13.88; *SD* = 0.30 and *M* = 8.25; *SD* = 0.32; *F*(1,23) = 316.84, *p* < 0.001, $\eta_{p}^{2}$ = 0.93].

### Body Temperature

The Lighting (blue-enriched light and dim light) × Exercise-Induced Activation (high arousal and low arousal) ANOVA on skin temperatures (distal, proximal, and the DPG) during the PVT performance did not show significant results. Only the main effect of *activation* was marginally significant on the DPG measure, *F*(1,23) = 3.69, *p* = 0.07, $\eta_{p}^{2}$ = 0.14, suggesting that high activation might decrease the DPG temperature. The interaction between *lighting and activation* was far from significant in all temperature measures (*Fs* < 1).

### Auditory Psychomotor Vigilance Task (PVT)

**FIGURE 5:**
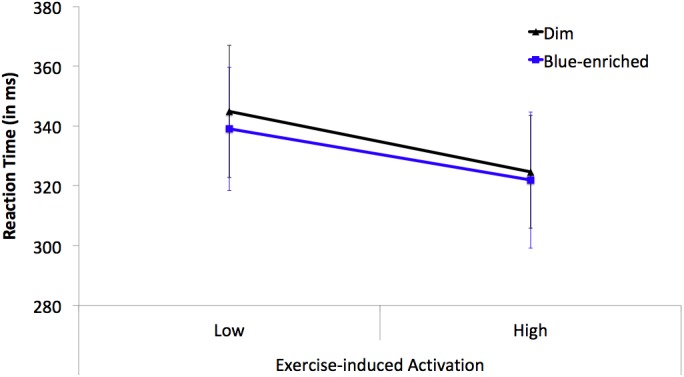
Mean reaction times (RTs) on the PVT as a function of Lighting (black line triangle marker: dim light; blue line square marker: blue-enriched light) and Exercise-induced Activation (low vs. high arousal). Vertical bars represent the standard error of the mean.

The Lighting (blue-enriched light and dim light) × Exercise-Induced Activation (high arousal and low arousal) ANOVA on the mean RTs from the PVT only showed a significant main effect of *activation*, *F*(1,23) = 8.52, *p* = 0.01, $\eta_{p}^{2}$ = 0.27, *B*_01_ = 0.01, with faster responses in the high arousal (*M* = 323 ms; *SD* = 13.97) than low arousal conditions (*M* = 342 ms; *SD* = 14.16). The main effect of lighting (*F* < 1, *B*_01_ = 4.59) and the lighting × activation (*F* < 1, *B*_01_ = 0.22, Figure 5) interaction were both far from significant.

The ANOVA on the inverse of RT replicated the findings on mean RTs [main effect of activation, *F*(1,23) = 18.34, *p* < 0.001, $\eta_{p}^{2}$ = 0.44, *B*_01_ = 0.002]; no main effect or interaction involving lighting, all *ps* > 0.26. The ANOVA on lapses revealed no statistically significant effects or interactions (all *ps* > 0.21).

## Discussion

This experiment tested whether a parametric manipulation of basal arousal induced by physical exercise would modulate the alerting effects of a blue-enriched white light on subjective (KSS, PANAS), physiological (skin temperature) and behavioral (PVT) measures, in a sample of 24 participants completing four sessions (high vs. low arousal × blue-enriched vs. dim light) in consecutive days at the beginning of the night (9–10 pm). According to previous correlational evidence (Correa et al., 2016) and research on caffeine and lighting (Wright et al., 1997; Beaven and Ekström, 2013), we expected that the combination of blue-enriched light and high arousal would produce the largest boosting of alertness.

The results showed that the exercise-induced manipulation of arousal produced robust alerting effects in most measures: attenuating subjective sleepiness (and enhancing affect), marginally decreasing the temperature gradient (DPG), and speeding responses in the PVT. In contrast, the lighting manipulation produced rather weak effects on alertness: attenuating subjective sleepiness (and incrementing positive affective balance), but not affecting behavior or physiology. Most relevant, the results did not confirm our hypothesis of an interaction between lighting and arousal, and Bayesian analyses further supported the null behavioral effects of our lighting manipulation.

Our finding that exercise-induced arousal speeded responses in the PVT replicated previous research using moderate-to-high intensity physical exertion (González-Fernández et al., 2017), validating the effectiveness of the current manipulation. Underlying this behavioral effect are multiple physiological processes triggered by physical exercise that influence the regulatory mechanisms of arousal: increase in catecholamines, HR, lactate, and hormones of the hypothalamo-pituitary-adrenergic axis (Borer, 2003).

On the other hand, lighting effects were most evident at the subjective level. Participants reported feeling less sleepy and having a positive affective balance when they were exposed to blue-enriched light as compared to dim light. The reduction of subjective sleepiness by lighting is consistent with the literature, as Souman et al. (2017) identified 28 out of 45 studies manipulating polychromatic light intensity that reported significant effects on this measure. In addition, we found that blue-enriched light selectively improved positive rather than negative affect, biasing the participants’ affective balance to the positive pole. This finding also replicates research about the effects of an acute exposure to blue lighting on emotion as measured by the PANAS (Plitnick et al., 2010; Sroykham and Wongsawat, 2013).

The acute effects of light on positive affect could be mediated by direct pathways between the photosensitive retinal ganglion cells (ipRGCs) and mood regulation centers like the amygdala and posterior hypothalamus (Vandewalle et al., 2010; LeGates et al., 2014), although it is also possible that participants’ beliefs and expectations on the positive effects of light might have influenced subjective measures (Veitch, 1997). This evidence was therefore complemented by physiological and behavioral measures.

The physiological effects on body temperature were only marginally observed in relation to the manipulation of physical exercise, such that the decrement of the DPG under the high intensity condition could be interpreted as an enhancement of alertness by counteracting the sleep promoting mechanism. Sleepiness has been associated with a reduction of core body temperature via peripheral vasodilation to dissipate heat through distal regions, and leading to a higher temperature gradient between distal and proximal sites (Kräuchi et al., 2000).

Lighting, however, did not influence skin temperature in this experiment, in contrast with many previous evidence (Badia et al., 1991; Cajochen et al., 2005; Lockley et al., 2006; Correa et al., 2016; Rodríguez-Morilla et al., 2017, 2018). The main difference between the abovementioned studies and the current one is that participants were at rest in the former while they were engaged in physical activity in all of our conditions. It is possible that a strong effect of physical activity on body temperature might have masked the subtle effect of lighting. To test this hypothesis, a resting condition would be necessary. However, we decided to discard this condition from our design, as it would add a confound related to the fact that the resting condition lacks the physical and mental demands of pedaling at a given cadence, which were more balanced in our two conditions of high vs. low intensity, where participants were always pedaling. Thus, our design prevented a confound regarding substantial differences in mental demands (and potential fatigue) between experimental and resting (control) conditions, which may be relevant for the effects of light (Smolders and de Kort, 2014). In other words, a resting condition would have been more sensitive to measure the effects of lighting on skin temperature, but it was not an adequate control condition for our main objective (testing whether the effect of blue-enriched lighting on vigilance performance depends on participants’ arousal level).

Finally, the main hypothesis of our study was not supported by the current findings. Our hypothesis of an interaction between lighting and physical activation can be derived from the Yerkes-Dodson law (Yerkes and Dodson, 1908): behavioral performance in an easy task like the PVT should be positively related to increments in arousal. We expected a summation of the increments in arousal based on the alerting effects of both lighting and physical exercise, which would lead to the fastest performance in the blue-enriched light plus high-arousal condition. This hypothesis was additionally supported by studies reporting faster RTs produced by a combined treatment of lighting and caffeine (Wright et al., 1997; Beaven and Ekström, 2013).

However, in our study only physical activity but not blue-enriched lighting improved response speed in the vigilance task. This null effect of lighting on behavior was further supported by Bayesian analyses. Moreover, the interaction between lighting and activation was far from significant in all PVT measures (mean RT, 1/RT, and lapses). Several complementary explanations might account for the current pattern of data.

First, it has already been mentioned that the low arousal condition may not be comparable to the resting condition typically used in other experiments finding a relationship between arousal and lighting effects (e.g., Correa et al., 2016). Hence, arousal increments in HR during the low arousal condition with respect to basal levels could have masked the subtler arousing effect of lighting. In other words, the arousing effect of physical exercise (even at low intensity) could have saturated the function of behavioral improvement predicted by the Yerkes-Dodson law. In any case, it can be concluded that the alerting effects are far more robust for physical exercise than for lighting.

It is also possible that lighting effects and their interaction with arousal are mainly observed in conditions of particularly low levels of arousal, such as under sleep deprivation (Wright et al., 1997; van Diepen et al., 2014) or mental fatigue (Smolders and de Kort, 2014). However, other studies showing lighting effects (and relation with arousal) in non sleep-deprived and non mentally-fatigued subjects (Beaven and Ekström, 2013; Correa et al., 2016) depart from this explanation.

Finally, other procedural differences across experiments (e.g., time of day, time of year, exposure duration, quality and quantity of lighting, etc.) might help explain the divergent results. It is further plausible that latitude differences across experimental settings could play a role on the robustness of lighting effects. People from southern or sunny countries like Spain could be more exposed to high levels of daylight than other northern locations traditionally leading research on lighting, therefore involving different photic history and sensitivity to the effects of light, in line with epidemiological data on the seasonal affective disorder showing higher prevalence at northern latitudes (Magnusson, 2000).

To conclude, the current study provides two fundamental contributions. At the theoretical level, we have tested a clear hypothesis stating that increasing the individuals’ basal level of arousal could enhance the behavioral effects of light. Our results did not confirm this hypothesis, and they suggest that acute exposure to a blue-enriched light is practically ineffective (only subjective reports were influenced) under conditions where the arousal level was over baseline. At the pragmatic level, the present research favored the use of acute physical exercise over acute exposure to blue-enriched lighting in order to boost human alertness when necessary. This exercise-related improvement has been shown to extend beyond the cessation of exercise to, at least, 20 min (Chang et al., 2012). However, since PVT performance was only measured while participants were pedaling in our study, future research should test the temporal dynamics of exercise-related improvements in vigilance.

This research provides implications for the use of acute physical exercise as an alertness enhancing factor in work settings where maintaining optimal levels of attention is difficult (shift work, night-work, vigilance tasks) and necessary to prevent human error and accidents.

## Author Contributions

FP, DS, ÁC, AB, and AL-C: Conceptualization, investigation, methodology, validation, and visualization. AB and ÁC: Data curation. AB, AL-C, and ÁC: Formal analysis. ÁC and DS: Funding acquisition and resources. ÁC and FP: Project administration. AB and AL-C: Software. ÁC, FP, and DS: Supervision. AB: Writing – original draft. ÁC, FP, DS, and AL-C: Writing, review, and editing.

## Conflict of Interest Statement

The authors declare that the research was conducted in the absence of any commercial or financial relationships that could be construed as a potential conflict of interest.

## References

[B1] Adan A. Almirall H. (1991). Horne and Östberg Morningness–eveningness questionnaire: a reduced scale. *Personal. Individ. Differ.* 12 241–253. 10.1016/0191-8869(91)90110-W

[B2] Akerstedt T. Gillberg M. (1990). Subjective and objective sleepiness in the active individual. *Int. J. Neurosci.* 52 29–37. 10.3109/00207459008994241 2265922

[B3] Badia P. Myers B. Boecker M. Culpepper J. Harsh J. R. (1991). Bright light effects on body temperature, alertness, EEG and behavior. *Physiol. Behav.* 50 583–588. 10.1016/0031-9384(91)90549-4 1801013

[B4] Beaven C. M. Ekström J. (2013). A Comparison of blue light and caffeine effects on cognitive function and alertness in humans. *PLoS One* 8:e76707. 10.1371/journal.pone.0076707 24282477 PMC3838207

[B5] Borer K. (2003). *Exercise Endocrinology.* Champaign, IL: Human Kinetics.

[B6] Borg G. (1970). Perceived exertion as an indicator of somatic stress. *Scand. J. Rehabil. Med.* 2 92–98. 5523831

[B7] Buysse D. J. Reynolds C. F. Monk T. H. Berman S. R. Kupfer D. J. (1989). The pittsburgh sleep quality index: a new instrument for psychiatric practice and research. *Psychiatry Res.* 28 193–213. 10.1016/0165-1781(89)90047-4 2748771

[B8] Cajochen C. Münch M. Kobialka S. Kräuchi K. Steiner R. Oelhafen P. (2005). High sensitivity of human melatonin, alertness, thermoregulation, and heart rate to short wavelength light. *J. Clin. Endocrinol. Metab.* 90 1311–1316. 10.1210/jc.2004-0957 15585546

[B9] Cajochen C. Zeitzer J. M. Czeisler C. A. Dijk D. J. (2000). Dose-response relationship for light intensity and ocular and electroencephalographic correlates of human alertness. *Behav. Brain Res.* 115 75–83. 10.1016/S0166-4328(00)00236-9 10996410

[B10] Chang Y. K. Labban J. D. Gapin J. I. Etnier J. L. (2012). The effects of acute exercise on cognitive performance: a meta-analysis. *Brain Res.* 1453 87–101. 10.1016/j.brainres.2012.02.068 22480735

[B11] Correa A. Barba A. Padilla F. (2016). Light Effects on Behavioural Performance Depend on the Individual State of Vigilance. *PLoS One* 11:e0164945. 10.1371/journal.pone.0164945 27820822 PMC5098788

[B12] Dinges D. F. Powell J. W. (1985). Microcomputer analyses of performance on a portable, simple visual RT task during sustained operations. *Behav. Res. Methods Instrum. Comput.* 17 652–655. 10.3758/BF03200977

[B13] Fisher J. P. (2014). Autonomic control of the heart during exercise in humans: role of skeletal muscle afferents. *Exp. Physiol.* 99 300–305. 10.1113/expphysiol.2013.074377 23995102

[B14] Gabel V. Maire M. Reichert C. F. Chellappa S. L. Schmidt C. Hommes V. (2015). Dawn simulation light impacts on different cognitive domains under sleep restriction. *Behav. Brain Res.* 281 258–266. 10.1016/j.bbr.2014.12.043 25549858

[B15] González-Fernández F. Etnier J. L. Zabala M. Sanabria D. (2017). Vigilance performance during acute exercise. *Int. J. Sport Psychol.* 48 435–447. 10.7352/IJSP2017.48.435

[B16] Horne J. A. Ostberg O. (1976). A self-assessment questionnaire to determine morningness-eveningness in human circadian rhythms. *Int. J. Chronobiol.* 4 97–110. 1027738

[B17] Huiberts L. M. Smolders K. C. de Kort Y. A. (2015). Shining light on memory: effects of bright light on working memory performance. *Behav. Brain Res.* 294 234–245. 10.1016/j.bbr.2015.07.045 26215575

[B18] Jarosz A. Wiley J. (2014). What are the odds? A practical guide to computing and reporting bayes factors. *J. Problem Solv.* 7. 10.7771/1932-6246.1167

[B19] Kräuchi K. Cajochen C. Werth E. Wirz-Justice A. (1999). Warm feet promote the rapid onset of sleep. *Nature* 401 36–37. 10.1038/43366 10485703

[B20] Kräuchi K. Cajochen C. Werth E. Wirz-Justice A. (2000). Functional link between distal vasodilation and sleep-onset latency? *Am. J. Physiol. Regul. Integr. Comp. Physiol.* 278 R741–R748. 10.1152/ajpregu.2000.278.3.R741 10712296

[B21] Lara T. Molina E. Madrid J. A. Correa A. (2018). Electroencephalographic and skin temperature indices of vigilance and inhibitory control. *Psicol. J.* 39 223–260. 10.2478/psicolj-2018-0010

[B22] LeGates T. A. Fernandez D. C. Hattar S. (2014). Light as a central modulator of circadian rhythms, sleep and affect. *Nat. Rev. Neurosci.* 15 443–454. 10.1038/nrn3743 24917305 PMC4254760

[B23] Lockley S. W. Evans E. E. Scheer F. A. Brainard G. C. Czeisler C. A. Aeschbach D. (2006). Short-wavelength sensitivity for the direct effects of light on alertness, vigilance, and the waking electroencephalogram in humans. *Sleep* 29 161–168. 10.1093/sleep/29.2.161 16494083

[B24] Lucas R. J. Peirson S. N. Berson D. M. Brown T. M. Cooper H. M. Czeisler C. A. (2014). Measuring and using light in the melanopsin age. *Trends Neurosci.* 37 1–9. 10.1016/j.tins.2013.10.004 24287308 PMC4699304

[B25] Magnusson A. (2000). An overview of epidemiological studies on seasonal affective disorder. *Acta Psychiatr. Scand.* 101 176–184. 10.1046/j.0902-4441.2000.x 10721866

[B26] Mann T. Lamberts R. P. Lambert M. I. (2013). Methods of Prescribing Relative Exercise Intensity: physiological and Practical Considerations. *Sports Med.* 43 613–625. 10.1007/s40279-013-0045-x 23620244

[B27] Molina E. Sanabria D. Jung T.-P. Correa A. (2017). Electroencephalographic and peripheral temperature dynamics during a prolonged psychomotor vigilance task. *Accident Anal. Prev.* 10.1016/j.aap.2017.10.014 [Epub ahead of print]. 29061281

[B28] Nobrega A. C. L. O’Leary D. Silva B. M. Marongiu E. Piepoli M. F. Crisafulli A. (2014). Neural regulation of cardiovascular response to exercise: role of central command and peripheral afferents. *BioMed Res. Int.* 2014:478965. 10.1155/2014/478965 24818143 PMC4000959

[B29] Ortiz-Tudela E. Martinez-Nicolas A. Campos M. Rol M. Á. Madrid J. A. (2010). A new integrated variable based on thermometry, actimetry and body position (TAP) to evaluate circadian system status in humans. *PLoS Comput. Biol.* 6:e1000996. 10.1371/journal.pcbi.1000996 21085644 PMC2978699

[B30] Plitnick B. Figueiro M. Wood B. Rea M. (2010). The effects of red and blue light on alertness and mood at night. *Light. Res. Technol.* 42 449–458. 10.1177/1477153509360887 23535242

[B31] Rodríguez-Morilla B. Madrid J. A. Molina E. Correa A. (2017). Blue-enriched white light enhances physiological arousal but not behavioral performance during simulated driving at early night. *Front. Psychol.* 8:997. 10.3389/fpsyg.2017.00997 28690558 PMC5479916

[B32] Rodríguez-Morilla B. Madrid J. A. Molina E. Pérez-Navarro J. Correa A. (2018). Blue-enriched light enhances alertness but impairs accurate performance in evening chronotypes driving in the morning. *Front. Psychol.* 9:688. 10.3389/fpsyg.2018.00688 29867659 PMC5962740

[B33] Romeijn N. Van Someren E. J. W. (2011). Correlated fluctuations of daytime skin temperature and vigilance. *J. Biol. Rhythms* 26 68–77. 10.1177/0748730410391894 21252367

[B34] Santhi N. Groeger J. A. Archer S. N. Gimenez M. Schlangen L. J. M. Dijk D.-J. (2013). Morning sleep inertia in alertness and performance: effect of cognitive domain and white light conditions. *PLoS One* 8:e79688. 10.1371/journal.pone.0079688 24260280 PMC3832615

[B35] Schneider W. Eschman A. Zuccolotto A. (2002). *E-Prime User’s Guide.* Pittsburgh, PA: Psychology Software Tools Inc.

[B36] Smolders K. de Kort Y. A. W. (2014). Bright light and mental fatigue: effects on alertness, vitality, performance and physiological arousal. *J. Environ. Psychol.* 39 77–91. 10.1016/j.jenvp.2013.12.010

[B37] Souman J. L. Tinga A. M. te Pas S. F. van Ee R. Vlaskamp B. N. S. (2017). Acute alerting effects of light: a systematic literature review. *Behav. Brain Res.* 337 228–239. 10.1016/j.bbr.2017.09.016 28912014

[B38] Sroykham W. Wongsawat Y. (2013). Effects of LED-backlit computer screen and emotional selfregulation on human melatonin production. *Conf. IEEE Eng. Med. Biol. Soc.* 2013 1704–1707. 10.1109/EMBC.2013.6609847 24110034

[B39] Tanaka H. Monahan K. D. Seals D. R. (2001). Age-predicted maximal heart rate revisited. *J. Am. Coll. Cardiol.* 37 153–156. 10.1016/S0735-1097(00)01054-8 11153730

[B40] van Diepen H. C. Lucassen E. A. Yasenkov R. Groenen I. Ijzerman A. P. Meijer J. H. (2014). Caffeine increases light responsiveness of the mouse circadian pacemaker. *Eur. J. Neurosci.* 40 3504–3511. 10.1111/ejn.12715 25196050

[B41] van Marken Lichtenbelt W. D. Daanen H. A. Wouters L. Fronczek R. Raymann R. J. Severens N. M. (2006). Evaluation of wireless determination of skin temperature using iButtons. *Physiol. Behav.* 88 489–497. 10.1016/j.physbeh.2006.04.026 16797616

[B42] Vandewalle G. Gais S. Schabus M. Balteau E. Carrier J. Darsaud A. (2007a). Wavelength-dependent modulation of brain responses to a working memory task by daytime light exposure. *Cereb. Cortex* 17 2788–2795. 10.1093/cercor/bhm007 17404390

[B43] Vandewalle G. Schmidt C. Albouy G. Sterpenich V. Darsaud A. Rauchs G. (2007b). Brain responses to violet, blue, and green monochromatic light exposures in humans: prominent role of blue light and the brainstem. *PLoS One* 2:e1247. 10.1371/journal.pone.0001247 18043754 PMC2082413

[B44] Vandewalle G. Schwartz S. Grandjean D. Wuillaume C. Balteau E. Degueldre C. (2010). Spectral quality of light modulates emotional brain responses in humans. *Proc. Natl. Acad. Sci. U.S.A.* 107 19549–19554. 10.1073/pnas.1010180107 20974959 PMC2984196

[B45] Veitch J. A. (1997). Revisiting the performance and mood effects of information about lighting and fluorescent lamp type. *J. Environ. Psychol.* 17 253–262. 10.1006/jevp.1997.0059

[B46] Warm J. S. Parasuraman R. Matthews G. (2008). Vigilance requires hard mental work and is stressful. *Hum. Factors* 50 433–441. 10.1518/001872008X312152 18689050

[B47] Watson D. Clark L. A. Tellegen A. (1988). Development and validation of brief measures of positive and negative affect: the PANAS scales. *J. Pers. Soc. Psychol.* 54 1063–1070. 10.1037/0022-3514.54.6.1063 3397865

[B48] Williamson A. Lombardi D. A. Folkard S. Stutts J. Courtney T. K. Connor J. L. (2011). The link between fatigue and safety. *Accident Anal. Prev.* 43 498–515. 10.1016/j.aap.2009.11.011 21130213

[B49] Wright K. P. Badia P. Myers B. L. Plenzler S. C. (1997). Combination of bright light and caffeine as a countermeasure for impaired alertness and performance during extended sleep deprivation. *J. Sleep Res.* 6 26–35. 10.1046/j.1365-2869.1997.00022.x 9125696

[B50] Yerkes R. M. Dodson J. D. (1908). The relation of strength of stimulus to rapidity of habit-formation. *J. Comp. Neurol. Psychol.* 18 459–482. 10.1002/cne.920180503

